# Identification of a genetic region linked to tolerance to MRSA infection using Collaborative Cross mice

**DOI:** 10.1371/journal.pgen.1011378

**Published:** 2024-08-23

**Authors:** Aravindh Nagarajan, Kristin Scoggin, L. Garry Adams, David Threadgill, Helene Andrews-Polymenis

**Affiliations:** 1 Interdisciplinary Program in Genetics and Genomics, Texas A&M University, College Station, Texas, United States of America; 2 Department of Microbial Pathogenesis and Immunology, Texas A&M University, College Station, Texas, United States of America; 3 Department of Veterinary Pathobiology, Texas A&M University, College Station, Texas, United States of America; 4 Department of Molecular and Cellular Medicine, Texas A&M University, College Station, Texas, United States of America; 5 Texas A&M Institute for Genome Sciences and Society, Texas A&M University, College Station, Texas, United States of America; 6 Department of Biochemistry & Biophysics and Department of Nutrition, Texas A&M University, College Station, Texas, United States of America; The Jackson Laboratory, UNITED STATES OF AMERICA

## Abstract

*Staphylococcus aureus* (*S*. *aureus*) colonizes humans asymptomatically but can also cause opportunistic infections, ranging from mild skin infections to severe life-threatening conditions. Resistance and tolerance are two ways a host can survive an infection. Resistance is limiting the pathogen burden, while tolerance is limiting the health impact of a given pathogen burden. In previous work, we established that collaborative cross (CC) mouse line CC061 is highly susceptible to Methicillin-resistant *S*. *aureus* infection (MRSA, USA300), while CC024 is tolerant. To identify host genes involved in tolerance after *S*. *aureus* infection, we crossed CC061 mice and CC024 mice to generate F1 and F2 populations. Survival after MRSA infection in the F1 and F2 generations was 65% and 55% and followed a complex dominant inheritance pattern for the CC024 increased survival phenotype. Colonization in F2 animals was more extreme than in their parents, suggesting successful segregation of genetic factors. We identified a Quantitative Trait Locus (QTL) peak on chromosome 7 for survival and weight change after infection. In this QTL, the WSB/EiJ (WSB) allele was present in CC024 mice and contributed to their MRSA tolerant phenotype. Two genes, *C5ar1* and *C5ar2*, have high-impact variants in this region. *C5ar1* and *C5ar2* are receptors for the complement factor *C5a*, an anaphylatoxin that can trigger a massive immune response by binding to these receptors. We hypothesize that *C5a* may have altered binding to variant receptors in CC024 mice, reducing damage caused by the cytokine storm and resulting in the ability to tolerate a higher pathogen burden and longer survival.

## Author summary

*Staphylococcus aureus* causes a wide range of life-threatening diseases in humans. Resistance and tolerance are two ways a host can survive an infection. Resistance is limiting the pathogen burden, while tolerance is limiting the health impact of a given pathogen burden. Tolerance mechanisms are poorly understood in context of host-pathogen interaction. To identify host genes involved in tolerance after S. aureus infection, we crossed CC061 mice and CC024 mice. The genetic factors controlling tolerance were well segregated in the F2 population. Using QTL mapping, we identified a significant peak on chromosome 7 for survival and weight change after infection. Two genes, C5ar1 and C5ar2, have high-impact variants in this region. We hypothesize that C5a may have altered binding to variant receptors in CC024 mice, reducing damage caused by the cytokine storm and resulting in the ability to tolerate a higher pathogen burden and longer survival.

## Introduction

*Staphylococcus aureus (S*. *aureus*) is a gram-positive coccus well-adapted to humans and a variety of companion, farm, and wild animals [1]. *S*. *aureus* is a commensal organism as well as an opportunist that can cause severe morbidity and mortality [2,3]. Globally, *S*. *aureus* is one of the leading causes of bacterial-related hospitalizations and is responsible for more than one million deaths annually [4]. *S*. *aureus* is a growing cause of concern because it spreads easily, and many isolates are resistant to several classes of antibiotics [5,6].

Nasal carriage of *S*. *aureus* varies widely across the human population [7,8]. Not all colonized individuals develop infections, but colonization is a risk factor for skin and soft tissue infections [9,10]. These superficial infections are precursors for 50% of *S*. *aureus* bacteremia (SAB) [11], and approximately one-third of SAB cases lead to sepsis [12,13]. This variability in disease outcomes after *S*. *aureus* colonization, suggests that this host-pathogen interaction may depend on both bacterial and host genetics.

Resistance and tolerance are two ways a host can survive an infection. Resistance is defined as maintaining health by limiting the pathogen burden, while tolerance is limiting the health impact of infection despite a high pathogen burden [14]. Mechanisms underlying tolerance to infection have recently been described for parasitic [15,16] and viral infections [17]. However, mechanisms underlying tolerance to bacterial infections are poorly understood [18].

The Collaborative Cross (CC) is a large panel of genetically diverse recombinant mouse strains derived from eight founder strains (five classical inbred strains—A/J, C57BL/6J (BL6), 129S1/SvlmJ, NOD/ShiLtJ, NZO/HlLtJ, and three wild-derived strains—CAST/EiJ, PWK/PhJ, and WSB/EiJ). This collection captures approximately 90% of the genetic diversity in laboratory mice [19–21]. With fully sequenced genomes, CC strains are ideal candidates for Quantitative Trait Locus (QTL) mapping [22,23]. Crosses between CC strains have been successful in identifying genetic loci underlying susceptibility to seizure [24], and *Mycobacterium tuberculosis* [25], and *Salmonella* Typhimurium [26] infections.

Host genetic variation plays role in sensitivity, tolerance and resistance mechanisms [27–30]. We created a large F2 intercross panel by crossing collaborative cross (CC) strains with known phenotypes with respect to MRSA USA300 infection, using a MRSA susceptible strain (CC061) and a tolerant strain (CC024). In our previous work we determined that parental CC061 mice rapidly lost weight and met our euthanasia criteria, while CC024 mice lost less weight and survived the study period [31]. Cytokine and gene expression patterns differed between these strains both before and after infection. In this work we demonstrate that survival in F1 and F2 generation followed a complex dominant pattern of inheritance for the CC024 phenotype. Colonization in F2 animals was more extreme than in the parental strains, suggesting successful segregation of genetic factors. We identified a QTL peak on chromosome 7 for survival and weight change after infection. Several genes located in this QTL region suggest a mechanism for tolerance to MRSA infection.

## Results

### CC061 and CC024 differ in survival after infection with MRSA USA300

**Fig 1:**
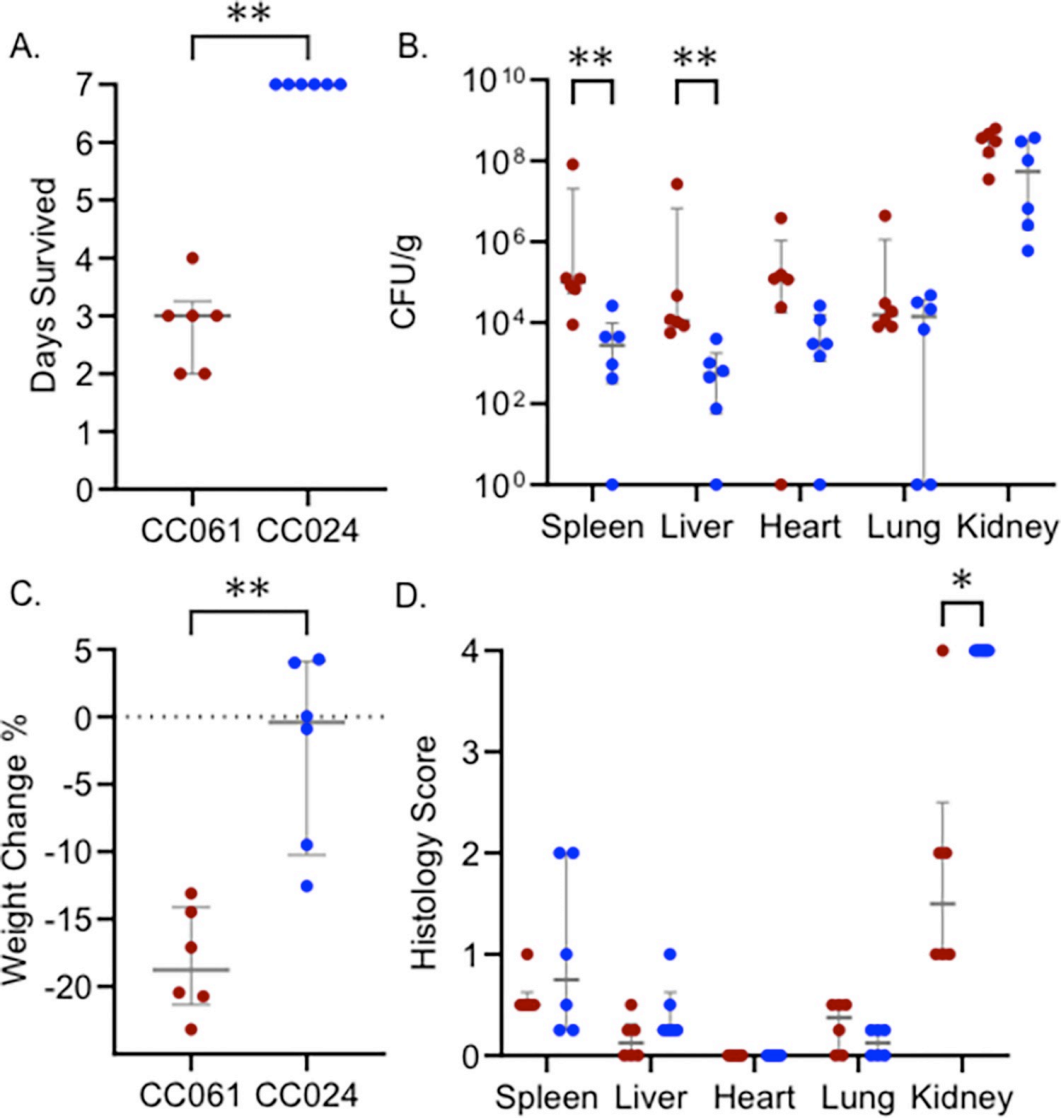
CC061 and CC024 differ in survival after infection with MRSA USA300. After intravenous infection with MRSA USA300 (underlying data from our previous work [31]) we show: A. Survival time, B. Colonization, C. Percent weight change after infection, and D. Tissue damage in the spleen, liver, heart, lung, and kidney. Dots represent individual mice; red dots represent CC061; blue dots represent CC024. The median and interquartile range are shown for each strain. Mann-Whitney test was performed to determine statistical significance (* = P < 0.05, ** = P < 0.01).

In our previous study, two CC strains, CC061 and CC024, were infected with MRSA USA300 and monitored for a week after infection [31]. All of the CC061 mice met our euthanasia criteria with a median survival of three days and were classified as susceptible (Fig 1A). All the CC024 mice survived until day 7 with high organ colonization and were classified as tolerant (Fig 1A and 1B). CC061 mice lost 18% of their body weight on average (Fig 1C). Weight loss in CC024 mice was variable, three animals gained weight and three lost weight, a mean of -2.5% of body weight was lost across CC024 mice (Fig 1C). Kidney, heart, and lung colonization did not significantly differ between CC061 and CC024 (Fig 1B). Spleen and liver colonization were higher in CC061 than in CC024 mice, yet there was no difference in tissue damage scores in these two strains for the spleen and liver (Fig 1D). Paradoxically, we observed more severe damage in the kidney in tolerant CC024 mice than in sensitive CC061 mice (Fig 1D). This finding contradicts the previous dogma that the ability to reduce tissue damage may be a feature of the tolerance phenotype [32,33].

### Immune profile differences between CC061 and CC024

**Fig 2:**
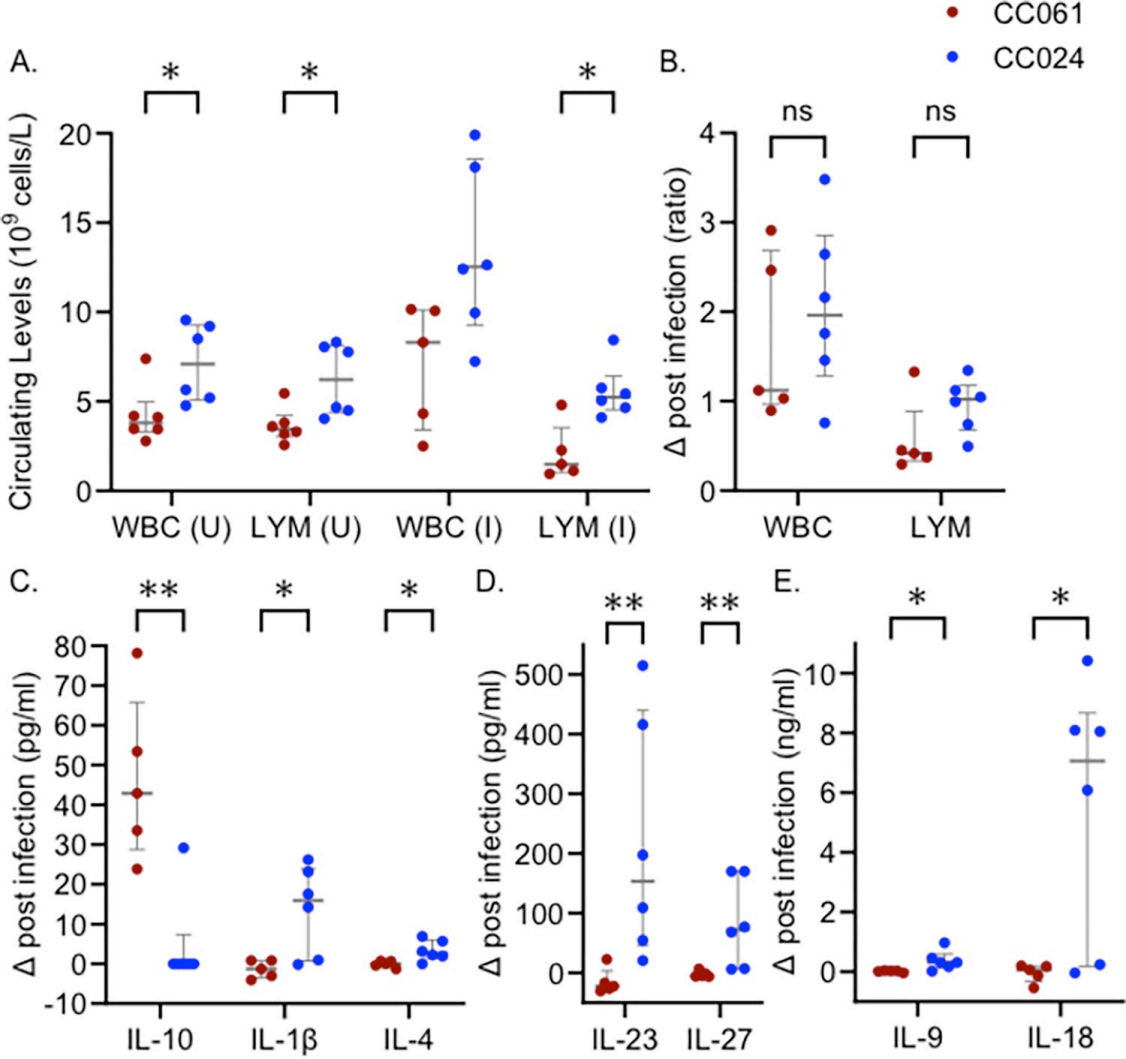
Immune differences between CC061 and CC024. A. Circulating levels of white blood cells (WBC) and lymphocytes (LYM) in uninfected (U) and infected (I) mice. B. Change in WBC and LYM levels post-infection. C-E. Change in chemokine levels post-infection. Dots represent individual mice; red dots represent CC061; blue dots represent CC024. The median and interquartile range are shown for each strain. Mann-Whitney test was performed to determine statistical significance (ns–no significance, * = P < 0.05, ** = P < 0.01).

Each CC strain has a unique immune profile [34,35], so we examined the differences in immune parameters between CC024 and CC061. CC024 mice have higher baseline circulating white blood cells (WBC) before infection than CC061 mice, driven by higher numbers of lymphocytes (LYM) (Fig 2A). After infection, both WBC (P = 0.51) and LYM were elevated in CC024 mice compared to CC061 mice (Fig 2A), but the change from baseline levels was similar in both lines (Fig 2B).

After accounting for strain differences in baseline levels of these cytokines, seven cytokines differ significantly between CC024 mice and CC061 mice post-infection. IL-10 was significantly increased in CC061 mice after MRSA infection (Fig 2C), while IL-1β, IL-4, IL-9, IL-18, IL-23, and IL-27 were significantly increased in CC024 mice after infection (Fig 2C, 2D and 2E). These differences in immune parameters suggest that the two strains differ in their immune response after infection.

### Survival is linked to dominant alleles in CC024 mice

**Fig 3:**
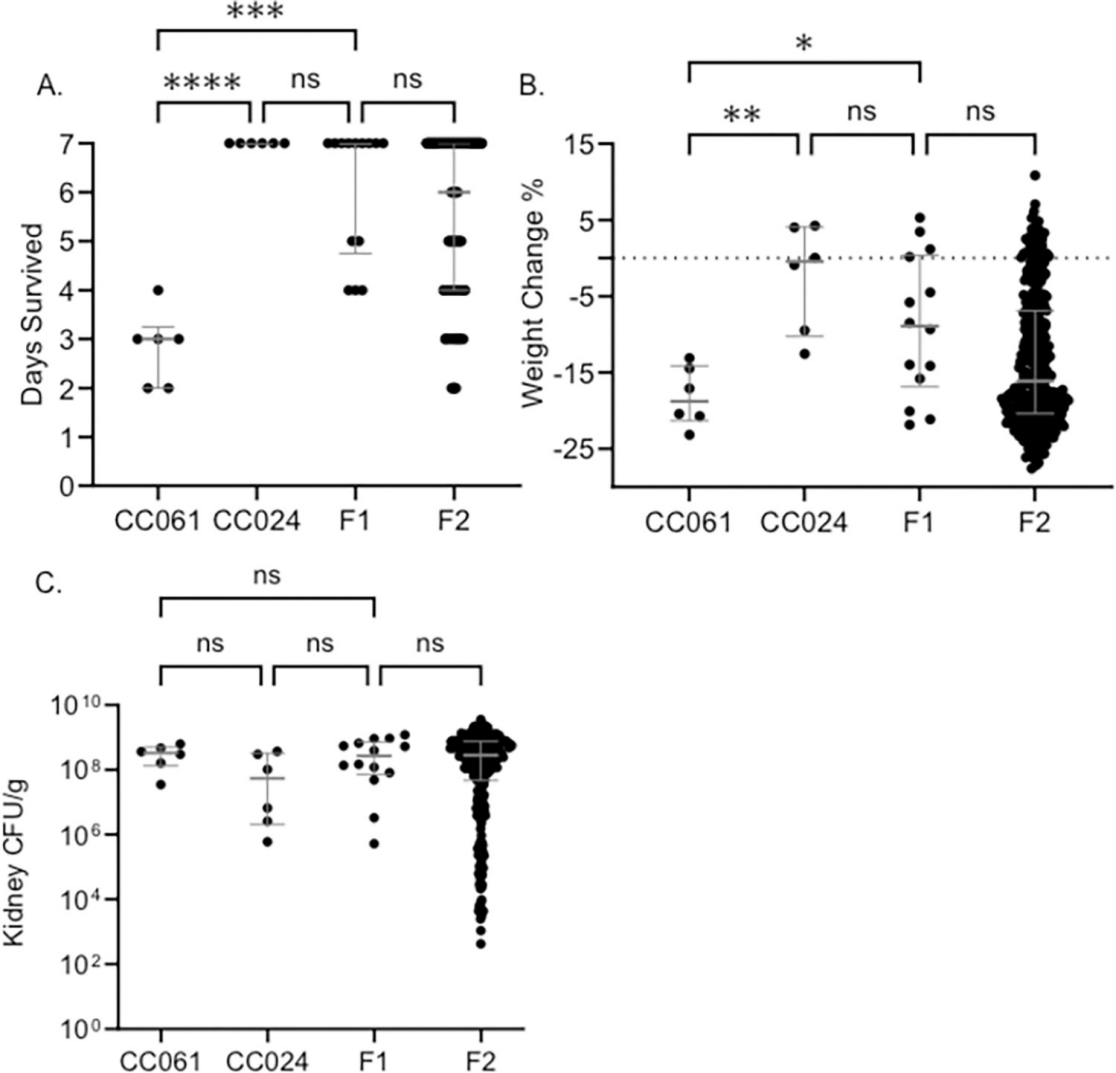
The F2 population is similar to F1 in survival and weight change after infection. A. Survival time B. Percent weight change after infection C. Kidney colonization. Dots represent individual mice. Significance tests were performed between the two parents and F1 and between F1 and F2. The median and interquartile range are shown. Kruskal-Wallis test was performed to test for significance (ns–no significance, * = P < 0.05, ** = P < 0.01, *** = P < 0.001, **** = P < 0.0001). Data for CC061 and CC024 parents is reproduced from our previous work for direct comparison to F1 and F2 generations.

To understand the genetic components involved in tolerance after infection, we created an F1 population by crossing MRSA susceptible CC061 mice with MRSA tolerant CC024 mice. We infected 14 animals from the F1 generation and compared their phenotypes with those of their parents. The median survival for the F1 generation was seven days, similar to CC024 mice and significantly higher than CC061 mice (Fig 3A). F1 mice maintained their weight more similar to the CC024 parent, most losing less weight than the CC061 parent (Fig 3B). These data support a complex dominant inheritance pattern for survival in the F1 cross between CC061 and CC024 mice. Survival after MRSA infection has a genetic component and is likely linked to dominant alleles in CC024.

### The F2 population is similar to the F1 generation in survival and weight change after MRSA infection

F1 mice were bred together to generate a large panel of F2 mice to further investigate the genetic components of tolerance and susceptibility. We infected 316 F2 mice with MRSA USA300. Among the 316 mice, 175 (55%) survived the 7-day study period, while the other 141 (45%) met our euthanasia criteria prior to day 7. Pre-infection weight (R = 0.04) and infection dose (R = 0.05) did not correlate with survival (S1A Fig). In the F2 population, weight change after infection (R = 0.67) and kidney colonization (R = -0.57) correlated significantly with survival (S1A Fig). Survival in the F2 population was not influenced by sex (S1B Fig). Weight change (P = 0.31) and kidney colonization (P = 0.61) was not significantly different between the males and females in the F2 population. The median survival for the F2 population was six days, very similar to the median survival of the F1 population (Fig 3A). Furthermore, the F1 and F2 generations were identical in weight change post-infection (Fig 3B). Kidney colonization was also similar between the F1 and F2 generations (Fig 3C). The similarity in infection outcome between the F1 and F2 indicates that the longer survival phenotype of the tolerant CC024 strain is dominant.

### Validation of genotype and QTL analysis using coat color

Using QTL mapping, we identified the genetic region, and ultimately strong candidate genes, mediating tolerance in the CC024 mice. Since CC024 and CC061 are fully homozygous, and their genomes are fully sequenced, we used the Mini Mouse Universal Genotyping Array (MiniMUGA) to genotype the F2 population. MiniMUGA has 11,000 markers that differentiate around 120 classical inbred lines and are equally spaced across the genome, enabling precise QTL mapping [36]. We used the coat color phenotype of our F2 population as a control for genotyping and QTL codes.

Our F2 population had two different coat colors: agouti and albino. These coat colors followed the Mendelian inheritance pattern of 3:1; 77% were agouti, and 23% were albino in our F2 population. We binary-coded (0 = agouti, and 1 = albino) the coat color data for our QTL analysis and identified a significant QTL peak on chromosome 7 (S2A Fig). The peak marker was located at 87.12 MB with a 1.5 Logarithm of Odds (LOD) drop range between 83 and 89 MB. CC061 allele and the heterozygote allele caused the agouti coat color, while the CC024 allele was responsible for the albino coat color (S2B Fig). Among the eight founders, A/J contributed the CC024 allele, and C57BL/6J contributed the CC061 allele in this region (S2C Fig).

We looked for variants in the founder alleles in this region with predicted deleterious effects on protein structure and function. We found a missense variant (rs31191169) in the A/J version of the Tyrosinase (*Tyr*) gene, changing amino acid 103 from cysteine to serine. The Sorting Intolerant from Tolerant (SIFT) score for this mutation is zero, suggesting this mutation strongly affects the Tyrosinase protein structure and function. Variants in the *Tyr* gene have previously been shown to influence coat color pigmentation in mice, including the CC [37,38]. The shortlisting of the *Tyr* gene variant using coat color is quality control for our other genotyping and QTL analyses.

### Genes on chromosome 7 control survival and weight change after infection in CC024 mice

**Fig 4:**
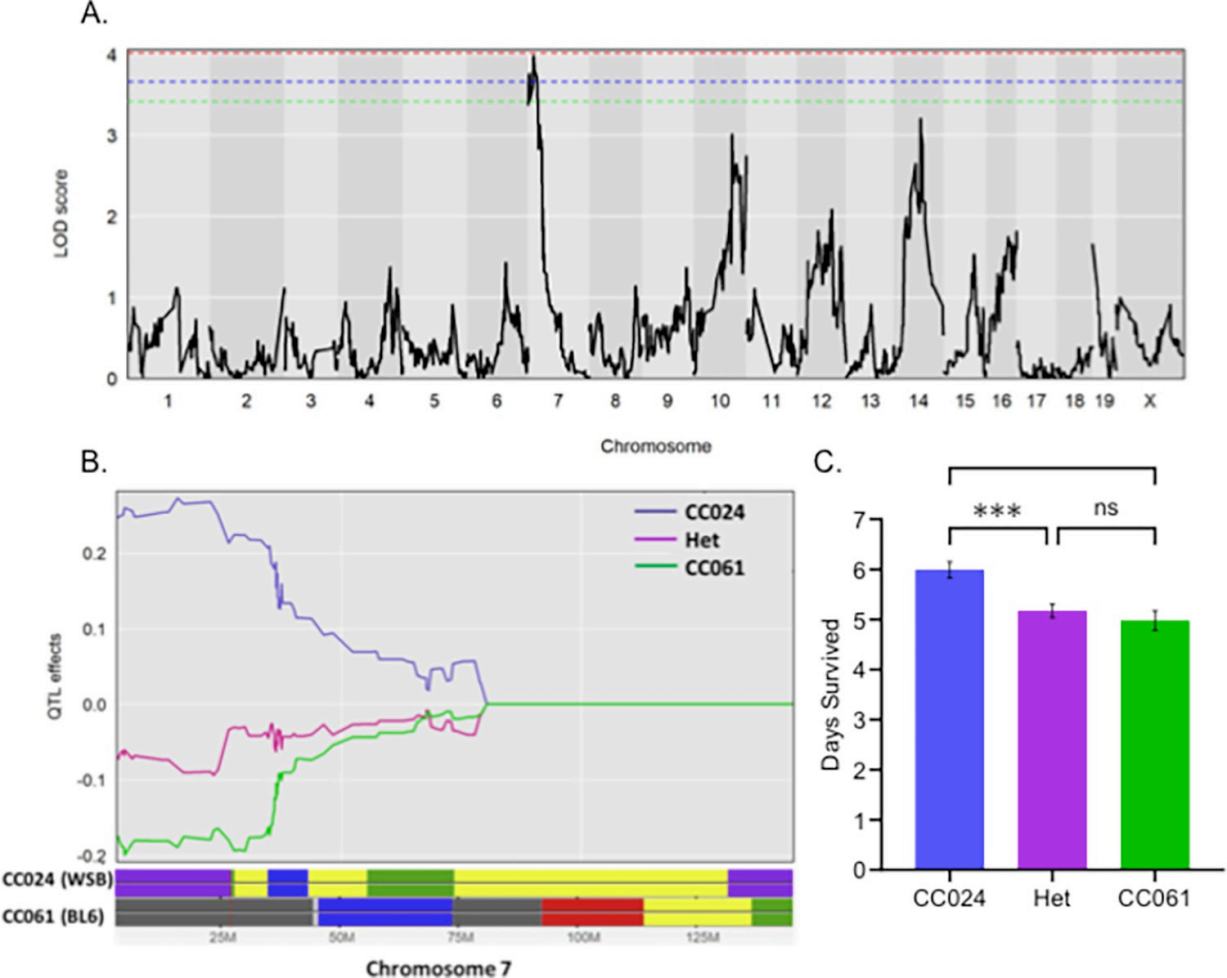
Survival after infection has a significant peak on chromosome 7. A. LOD plot for rank-transformed survival after infection (number of days). The dotted (Red– 95%, Blue– 90%, Green– 85%) lines represent the significant LOD scores for 999 permutations. B. Founder allele plot for chromosome 7. C. Founder allele contribution at the highest marker on chromosome 7, mean and standard error of the mean are shown. Het–Heterozygous allele for CC061 and CC024. Kruskal-Wallis test was performed to determine statistical significance (ns–no significance, *** = P < 0.001).

Survival, weight change, and colonization were considered complex traits for our QTL analysis using F2 mice. A significance threshold was established for individual phenotypes based on a permutation test [39]. A significant QTL peak on chromosome 7 was linked to survival (Fig 4A). We named this QTL peak 24SMI (CC024 Survival after MRSA Infection). The 1.5 drop confidence interval was between 4 and 25 MB, with a peak at 15.91 MB. As expected, the CC024 allele increased survival, while the CC061 and the heterozygous allele (CC061/CC024) reduced survival after infection (Fig 4B and 4C). In the confidence interval region, the WSB allele contributed to tolerance in CC024 mice, and the C57BL/6J allele contributed to the sensitivity phenotype of CC061 mice (Fig 4B). We identified a significant peak on chromosome 7 linked to weight change after infection (S3A Fig). We called this peak 24WMI (CC024 Weight change after MRSA Infection). The peak was located at 22.79 MB, with a confidence interval between 13 and 25 MB. This weight peak completely encompassed the survival peak and had the same founder effect pattern (S3B and S3C Fig). This result was not surprising as survival and weight change are very highly correlated (R = 0.67) (S1A Fig).

### Prioritizing variants on chromosome 7

**Fig 5:**
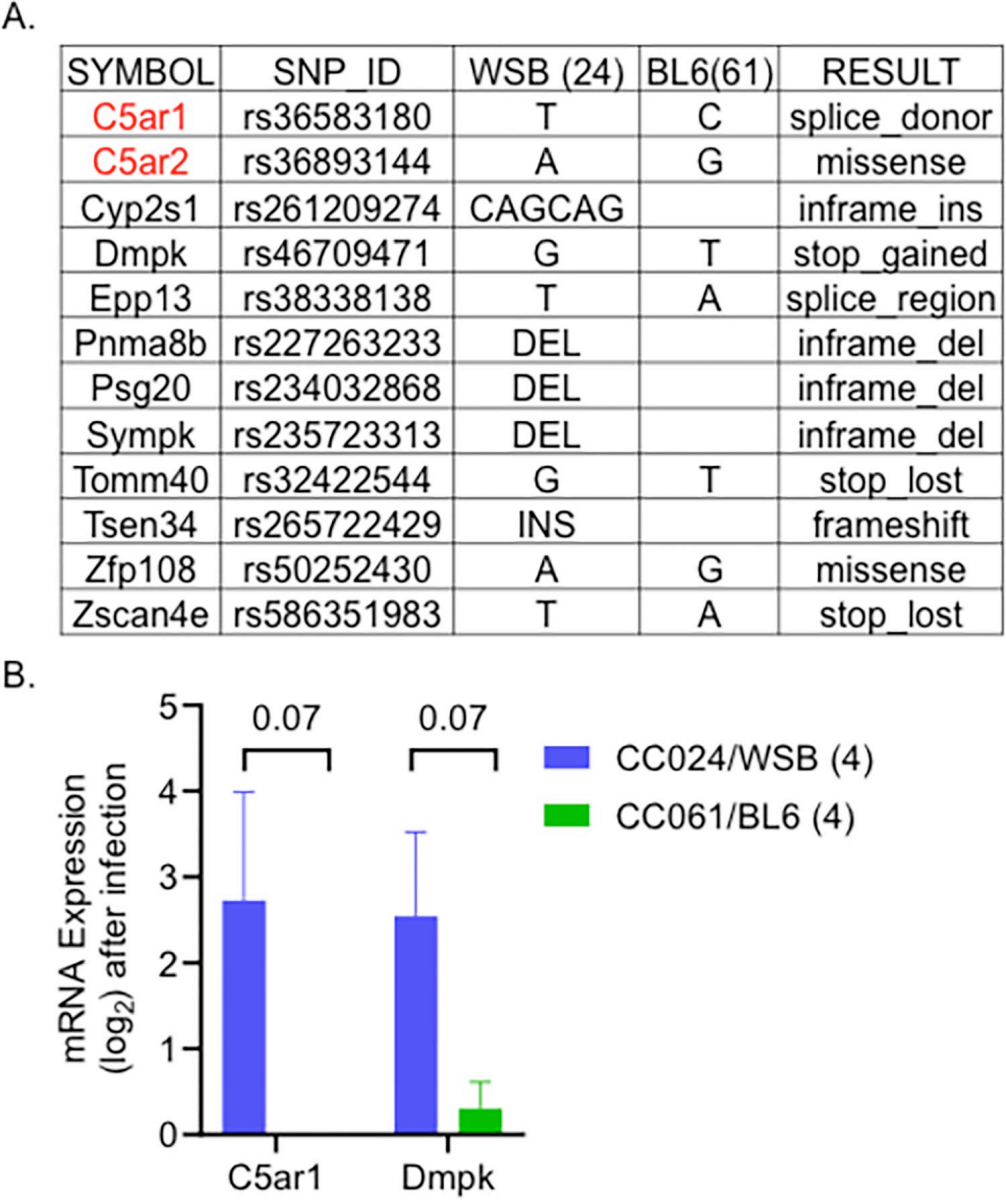
Prioritizing genes on chromosome 7 for survival after infection. A. Genes with high-impact variants shortlisted using the founder effect pattern. B. Kidney mRNA expression values after infection (log-transformed). Mean and standard error are shown. The number of animals is given in brackets. Mann-Whitney test was performed (Mean with standard error and P values are shown).

We used the Mouse Phenome Database (MPD) to prioritize variants (SNPs, insertions, or deletions) within our confidence interval linked to survival and weight loss. This 21 MB region has ~35,000 variants between the WSB and C57BL/6J alleles. We used Variant Effect Predictor (VEP) to shortlist variants within genes that were predicted to significantly impact protein structure and function. We identified 12 genes in this region with predicted high-impact variation on the structure and/or function of the predicted protein (S3 Table). The variant with the highest impact for each gene and previous immune system process associations in the Mouse Genome Informatics (MGI) database are shown in Fig 5A (highlighted in red). Using the total RNA sequencing data from the kidney of F2 mice, we further prioritized these high impact variants.

The mRNA expression levels of these 12 genes were not significantly different between F2 animals with a WSB and C57BL/6J allele at the peak location. This finding may be due to a low number of samples (four mice per group), or these variants may not affect transcript abundance. The closest variants to significance were Complement Component 5a Receptor 1 (*C5ar1*) and Dystrophia Myotonica-Protein Kinase (Dmpk) genes, with P-values of 0.07. Both genes were expressed more highly in F2 animals having WSB allele compared to the C57BL/6J allele at the QTL peak (Fig 5B). Among the shortlisted genes, both *C5ar1* and *C5ar2* are essential genes in the complement cascade and are involved in the immune response to infections [40]. None of the other candidate genes have any previously associated immune system function in the MGI database. Thus, based on previous associations with the immune function, enrichment of complement cascade in CC024 mice and F2 kidney mRNA expression data, *C5ar1* and *C5ar2* are our top candidate genes for involvement in the tolerance phenotype in the 24SMI (survival) QTL peak. The *C5ar1* gene in CC024 mice has a splice donor variant that can disrupt RNA splicing resulting in an altered protein sequence and structure. Similarly, *C5ar2* in CC024 mice has a predicted high-impact missense variant with a SIFT score of 0.03. The modified versions of *C5ar1* and *C5ar2* in CC024 mice contributed by the wild-derived WSB founder may provide a survival advantage relative to the C57BL/6J allele in CC061 mice.

### Gene expression differences between CC061 and CC024

**Fig 6:**
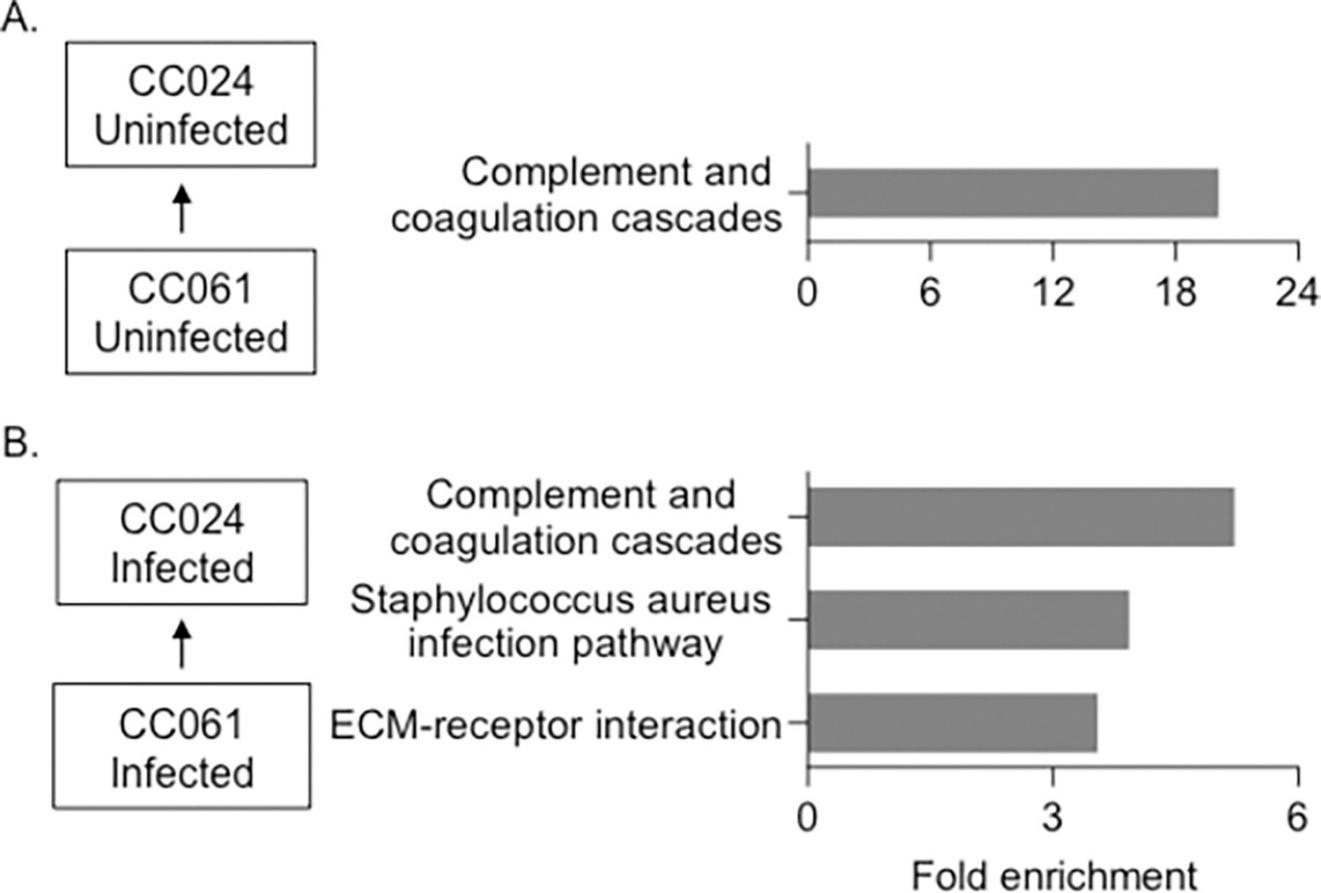
Gene expression differences between CC061 and CC024. Enriched pathways in CC024 compared to CC061 A. Before infection B. After infection. Fold enrichment was calculated by using all expressed genes as background.

We sequenced total RNA from kidneys (three males and three females) from CC061 and CC024 mice after infection to identify differences in gene expression. We also sequenced total RNA from age and sex-matched uninfected CC061s and CC024s to identify baseline differences. We compared differentially expressed genes between the two strains and looked for enriched KEGG pathways (KEGG- Kyoto Encyclopedia of Genes and Genomes). Prior to infection, the complement and coagulation cascades were the only pathways up regulated in CC024 mice compared to CC061 mice (Fig 6A). Complement genes, *C3* and *C4b*, and serine/cysteine peptidase inhibitors 1a and 1d contributed to this 20-fold enrichment. The complement system plays an important role in protecting the host against bacterial infections, including *S*. *aureus* [41,42]. Up-regulation of the complement cascade before and after infection in CC024 may play an important role in survival of CC024 mice after infection.

Three pathways were up regulated in CC024 mice compared to CC061 mice after MRSA infection (Fig 6B). In KEGG, the *S*. *aureus* infection pathway is part of the complement and coagulation cascade. Some notable genes that enriched these two pathways include complement components–*C1qa*, *C1qb*, *C1qc*, and *Cfh*, coagulation components–*Fgb*, *F2*, *F12*, *and Plg*, and serine/cysteine peptidase inhibitors *Serpina (1a-e and 2)* (S4 Table). The third pathway, extracellular matrix (ECM), contained genes, Fibronectin (*Fn1*), Vitronectin (*Vtn*), and collagens–*Col1a1*, *Col1a2*, and *Col6a3*.

## Discussion

Host genetics plays a major role in protection against bacterial infections [43]. Infection outcome in mice is also heavily dependent on the strain of inbred laboratory mice used, suggesting strong influence of host genetics [44]. Mice lacking Nod2 (Nucleotide-binding oligomerization domain-containing protein 2), Myd88 (Myeloid differentiation primary response protein), and Tlr2 (Toll-like receptor 2) are more susceptible to infection with *S*. *aureus* than mice expressing these genes [45,46]. We investigated the genetic factors behind the tolerance to infections with MRSA USA300. We chose a susceptible strain (C0061) and a tolerant strain (CC024) from our previous 7 day MRSA infection screen to identify genomic regions that are involved in these phenotypes [31]. The tolerant strain, CC024, survived longer and lost less weight than CC0061, despite similar colonization, including high colonization in the kidney.

After accounting for baseline strain differences, several serum cytokines differed between CC061 and CC024 mice post infection (Fig 2C, 2D and 2E). IL-10 is the only cytokine that was increased in the serum of CC061 mice. IL-10 is an anti-inflammatory cytokine produced by both innate and adaptive immune cells, and is a major immunoregulatory cytokine for modulating the production of several other cytokines [47–49]. Overexpression of IL-10 worsens the outcome of bacterial infections, including *S*. *aureus* infections [49]. IL-10 creates an anti-inflammatory environment by inhibiting the production of several pro-inflammatory cytokines and chemokines [50]. IL-10 inhibits T-helper cell responses [51], and down regulates MHC class II expression [52]. IL-10 also down regulates co-stimulatory molecules on antigen-presenting cells [53], further decreasing T-cell activation [54]. The increased production of IL-10 by CC061 mice may create an anti-inflammatory state that contributes to reduced survival of these mice during MRSA infection. Whether *S*. *aureus* itself manipulates IL-10 levels to evade the innate immune system is not clear.

Six cytokines were increased after infection in serum of MRSA tolerant CC024 mice, after accounting for pre-infection baseline differences. IL-1β and IL-18, elevated in CC024 mice after infection, act on lymphoid cells and enhance the production of IL-22 [55,56]. In addition, IL-1β also inhibits the production IL-10 by memory TH17 cells [57], counteracting the anti-inflammatory effects of IL-10. In *S*. *aureus* skin infections, both IL-4 and IL-23 (elevated in CC024) promote the production of IL-17 [58,59]. IL-27 is a pleiotropic cytokine that has both pro- and anti-inflammatory properties [60,61]. Though IL-27, along with IL-10, has been shown to enhance nasal colonization and increase susceptibility to *S*. *aureus* pneumonia [62,63], its role in suppressing inflammation in the later stages of infection may be beneficial in CC024 mice [60]. Overall, decreased IL-10, may promote survival of CC024 mice after MRSA infection. In a recent *S*. *aureus* vaccination study, increased levels of IL-10 and reduced IL-17 production lowered the efficiency of the vaccine [64], further supporting important roles for IL-10 in disease outcome after *S*. *aureus* infection.

We determined that the complement and coagulation cascade pathway is up regulated in CC024 mice both before and after MRSA infection. The complement system is one of the first host defense systems that *S*. *aureus* encounters during infection [42]. Complement consists of more than 30 proteins in plasma that opsonize bacteria, depositing complement activation products on the bacterial surface and attracting immune cells [65]. Complement can be activated by three pathways, which converge at the formation of *C3* convertase that cleaves *C3* into C3a and C3b [66]. C3b opsonizes *S*. *aureus*, while C3a activates immune cells, including macrophages and T cells [42]. *C3* deficient mice are more susceptible to *S*. *aureus* infections [67,68]. While we do not understand the mechanism underlying the baseline up-regulation of complement genes, *C3* and *C4b* in CC024, increased expression of these immune factors may improve CC024 survival by reducing the initial pathogen load.

To identify the genes needed for CC024 mice to tolerate MRSA infection despite high colonization in some organs, we generated an F2 cross between CC024 and CC061. Our data suggest that the genetic factors influencing MRSA colonization are well segregated in the F2 generation, and a complex trait controls their interaction. Survival in F2 mice ranged from 2 to 7 days post-infection, with a median of 6 days. Kidney colonization in F2 animals was roughly equivalent, irrespective of the euthanizing day. Thus, most of the F2 mice appear to be tolerant, as they survive the infection with higher bacterial loads (Fig 4). Survival correlated with kidney colonization (R = -0.57) and was considered a complex trait for QTL mapping.

The top candidate genes for the survival and weight change QTL in our analysis are the complement factor *C5a* receptors, *C5ar1* and *C5ar2*. In the earlier stages of infection, activation of the complement system is important to clear the pathogen [41]. However, during sepsis and later stages of infection, over-activation of complement negatively impacts the immune system [69]. Among the complement factors, *C5a* is the most potent inflammatory mediator to increase both pro-inflammatory factors, including TNF-α, IL-1β, IFN-γ, IL-6, and IL-8 and anti-inflammatory factors, including IL-10, IL-13, IL-4 and TGF-β [70–74]. Activation of the complement system leads to the cleavage of C5 into *C5a* and C5b [75]. *C5a*, which binds both *C5ar1* and *C5ar2*, is an anaphylatoxin and a chemotactic factor triggering an inflammatory response [76,77].

In mice and humans, *C5ar1* and *C5ar2* share high sequence identity but differ significantly in important regions that negatively affect the function of *C5ar2* [78]. Conflicting evidence exists on the potential roles of *C5ar2*: is it a decoy receptor to *C5ar1* with anti-inflammatory properties, or a unique signaling receptor with pro- or anti-inflammatory properties [79]? *C5ar1* is expressed in both myeloid cells, including macrophages, monocytes, and neutrophils and non-myeloid cells, including Kupffer cells, astrocytes, endothelial cells, and kidney tubular epithelial cells [80–84]. During sepsis, expression of *C5ar1* is up regulated in organs, including the lung, liver, heart, and kidney [85]. Although C5Ra1 and C5Ra2 are targets or receptors for staphylococcal virulence factors Chemotaxis Inhibiting Protein of *S*. *aureus* (CHIPS), Panton Valentin Leukocidin (PVL) and gamma hemolysin CB (hlgCB) in humans, murine neutrophils are resistant to PVL and hlgCB and do not bind CHIPS [86–91].

*C5ar* deficient mice have increased survival in cecal ligation and puncture (CLP) induced sepsis [92] and increased resistance to bacteremia and endotoxic shock [93]. Similarly, a *C5ar1* antagonist increased survival in CLP-induced sepsis [94] and reduced bacterial counts in several organs [85]. These data suggest that activation of *C5ar1* hastens the progression of sepsis. Binding of *C5a* to both *C5ar1* and *C5ar2* promotes inflammation and tissue damage [95–97]. Binding of *C5a* to these receptors results in increased production of several cytokines, chemotaxis of immune cells, intracellular calcium release, and superoxide anion production [40,98] that results in a cytokine storm damaging vital organs [99].

In CC024 mice, *C5ar1* and *C5ar2* have mutations that are predicted to significantly alter the structure of these *C5a* receptor proteins (Fig 6A). *C5a* may be either unable to or bind differentially to, the altered *C5ar1* and *C5ar2* receptors in CC024 mice, reducing inflammation, reducing the cytokine storm, reducing the resulting tissue damage, and potentially leading to tolerance. This hypothesis is also consistent with our finding that heterozygotes at these loci survive poorly (Fig 5B and 5C); a single CC061 allele for one of these receptors may be sufficient to allow activation of the complement system, leading to reduced survival in heterozygotes. These *C5ar1* and *C5ar2* variants, independently or together, are strong candidates for an important role in the increased survival during MRSA infection of CC024 mice.

Successfully eliminating a pathogen while minimizing damage to host tissue requires timing and coordination of the host immune system. Pathogens have evolved to take advantage of imperfections in the immune system. Here, natural variants occurring within the complement system could benefit the host by reducing inflammation and tissue damage in certain situations, thereby increasing the ability of the host to tolerate an infection for longer periods of time. With growing interest in targeting the complement system for developing therapeutics against sepsis [100–102], how natural variants of the complement system influence the effects of this system may be a fruitful avenue of future study.

## Materials and methods

### Ethics statement

All mouse studies followed the Guide for the Care and Use of Laboratory of Animals of the National Institutes of Health. The animal protocols (2015–0315 D and 2018–0488 D, 2019–0411) were reviewed and approved by Texas A&M Institutional Animal Care and Use Committee (IACUC).

### Bacterial strains and media

Methicillin-resistant *Staphylococcus aureus* isolate (MRSA USA300) used in this study was the kind gift of Dr. Magnus Hook (Texas A&M Institute of Biosciences and Technology, Houston). USA300 is a fully virulent, community-acquired clone of MRSA. Strains were routinely cultured in Luria-Bertani (LB) broth and plates supplemented with antibiotics when needed at 50 mg/L Kanamycin Sulphate. For murine infections, strains were grown aerobically at 37°C to stationary phase in LB broth supplemented with kanamycin. Stationary phase cultures were pelleted and resuspended in sterile LB broth for inoculation.

### Parental CC strains and data

Data for the two parental CC strains, were collected from a 1-week experiment performed previously, where CC024 and CC061 were part of a much larger screen of phenotypes of MRSA infected CC strains [31]. Data from this earlier study for CC024 and CC061 includes survival after infection, organ colonization, tissue damage after infection, weight change, complete blood count, and cytokine/chemokine levels (S2 Table) [31]. Tissue damage was evaluated in a blinded fashion by a board certified pathologist and rated on a scale of 0–4 (0 = no damage, 4 = severe damage) as previously described [31]. Complete blood counts (CBC) were performed on blood collected in EDTA tubes and evaluated on an Abaxis VetScan HM5 optimized for rodents [31].

We display this data here again, for easy comparison with F1 and F2 generation data generated in this work.

### Breeding and genotyping F1 and F2 generations

Collaborative Cross (CC) mouse strains–CC061(B) and CC024 (A), F1s ((A*B), (B*A)) and F2s ((AB*BA), (BA*AB), (AB*AB), (BA*BA)) were used for these experiments (S1 Table). The CC strains were initially purchased from UNC’s Systems Genetics Core Facility (SGCF) and were bred at the Division of Comparative Medicine at Texas A&M University to generate F1 and F2 generation (S1 Table). Mice were bred in trios (2 females and one male), and offspring were weaned at 21 days. Tail snips were collected during weaning and genotyped using the Mini MUGA panel at Neogen [36]. Mice were fed Envigo Teklad Global 19% Protein Extrudent Rodent Diet (2919) and were provided with cardboard huts, food, and water ad libitum.

### MRSA infection

After baseline scoring for health, F1 and F2 mice were injected with MRSA USA300. Briefly, Mice were anesthetized using isoflurane and injected with 1 x 10^7^ CFU in 50 μl of LB broth intravenously at the inferior fornix into the retro-orbital sinus. Mice that became moribund within 6 hours of infection were humanely euthanized and removed from the experiment. F2 mice were infected in 13 different batches over a 3-month period.

### Health monitoring

For F1 and F2 mice, we manually scored health parameters twice daily: physical appearance, body conditioning, and provoked and unprovoked behavior. The scoring scale ranged from 0–3, with zero = normal and three = abnormal. Additionally, as a measure of activity, four small nestlets were placed at each corner of the cage in the evening. The following day, the number of nestlets moved from the cage corners into the hut was recorded.

### Bacterial load determination

After infection, mice were monitored twice daily using visual health scoring. Mice that developed illness as measured by the health score were humanely euthanized by CO_2_ asphyxiation. After euthanasia, kidneys were collected. A consistent region of the right kidney was collected in 3mL ice-cold PBS and homogenized. The serially diluted homogenate was plated on LB plates supplemented with kanamycin for bacterial enumeration. Data are expressed as CFU/g of tissue.

### QTL analysis

Data from 315 F2 mice were included in the analysis. QTL analysis was performed using R/qtl2 software [103]. This method accounts for the complex population structure in CC strains. Briefly, MiniMuga genotypes (from tail snips) were imputed into the analysis. Monomorphic markers were removed. Genome scans were performed on the rank-transformed phenotype. The generated Logarithm of Odds (LOD) score is the likelihood ratio comparing the hypothesis of a QTL at a given position versus that of no QTL. Each phenotype was randomly shuffled 999 times to establish genome-wide significance, and LOD scores were calculated for each iteration [39]. The 85th percentile of the scores was considered significant for that phenotype. The genomic confidence interval was calculated by dropping the LOD scores by 1.5 for each significant peak. Mouse Genome Informatics (MGI) was used to find the genes and QTL features within each interval [104,105]. To further shortlist candidate genes, the founder strain distribution pattern was queried against the CC variant database (V3 Version) [106]. The Variant effect predictor (VEP) from the ensemble was used to calculate the impact score for the variant [107].

## RNA extraction and sequencing

RNA extraction and sequencing was performed as previously described [31]. Briefly, total RNA was extracted from frozen tissues using Direct-zol RNA Miniprep plus kit following the manufacturer’s protocol (Zymo Research—R2073). The purity and quantity of the extracted RNA were analyzed using RNA 6000 Nano LabChip Kit and Bioanalyzer 2100 (Agilent CA, USA, 5067–1511). High-Quality RNA samples with RIN number > 7.0 were selected for library construction. Following the vendor’s recommended protocol, we performed the 2×150bp paired-end sequencing (PE150) on an Illumina Novaseq 6000.

### RNA seq data analysis

Reads were trimmed using trim galore (version 0.6.7) [108]. This procedure removed adapters, poly tails, more than 5% of unknown nucleotides, and low quality reads containing more than 20% of low-quality bases (Q-value <20). Both forward and reverse hard trimmed at 100 base pairs. FastQC was used to verify data quality before and after cleaning [109]. Cleaned reads were aligned and counted against the mouse reference genome (GRCm39) using STAR (version 2.7.9a) aligner [110]. Downstream processing of the data was performed using IDEP 1.0 [111,112]. Gene counts were analyzed for differentially expressed genes using DESeq2 [113].

### Statistical methods

Most of the data collected for this study was not normally distributed. Thus, when comparing two groups, we used the Mann-Whitney (non-parametric) test to determine statistical significance. When three or more groups were compared, we used the Kruskal Wallis (non-parametric) test. Symbols in the figures correspond to the following P-values (ns–no significance, * = P < 0.05, ** = P < 0.01, *** = P < 0.001, **** = P < 0.0001).

## Supporting information

S1 Fig Infection outcome in F2 cross. A. Heat map showing Spearman correlation ‘R’ values between survival, weight change, Infection dose, and kidney CFU. B. Survival after infection separated by males and females. C. Kidney colonization of F2 mice by days survive. The median and interquartile range are shown. B–Mann-Whitney and C–Kruskal-Wallis tests were performed (ns- no significance, *** = P < 0.001).
(TIFF)

S2 Fig QTL for coat color in the F2 population. A. LOD plot for coat color after infection (0 –Agouti, 1 –Albino/white). The dotted (Red– 95%, Blue– 90%, Green– 85%) lines represent the significant LOD scores for 999 permutations. B. Founder allele plot for chromosome 7. C. Founder allele contribution for chromosome 7.
(TIFF)

S3 Fig QTL for weight change after infection in the F2 population. A. LOD plot for rank-transformed weight change after infection. The dotted (Red– 95%, Blue– 90%, Green– 85%) lines represent the significant LOD scores for 999 permutations. B. Founder allele plot for chromosome 7. C. Founder allele contribution for chromosome 7.
(TIFF)

S1 Table Mouse strains used in this study. Number of mice, origin, and location of breeding for all the mice used in the study.
(XLSX)

S2 Table Data associated with each mouse. All the pre-infection and post-infection data were associated with each mouse used in this study.
(XLSX)

S3 Table Shortlisted variants for QTL peak on chromosome 7 for survival after infection. (XLSX)

S4 Table Enriched KEGG pathway genes. (XLSX)
