# Supplementary figures and images for: Identification of a genetic region linked to tolerance to MRSA infection using Collaborative Cross mice
Source: PLoS Genet. 2024 Aug 23;20(8):e1011378. doi: 10.1371/journal.pgen.1011378 (PMC11407622; doi:10.1371/journal.pgen.1011378)

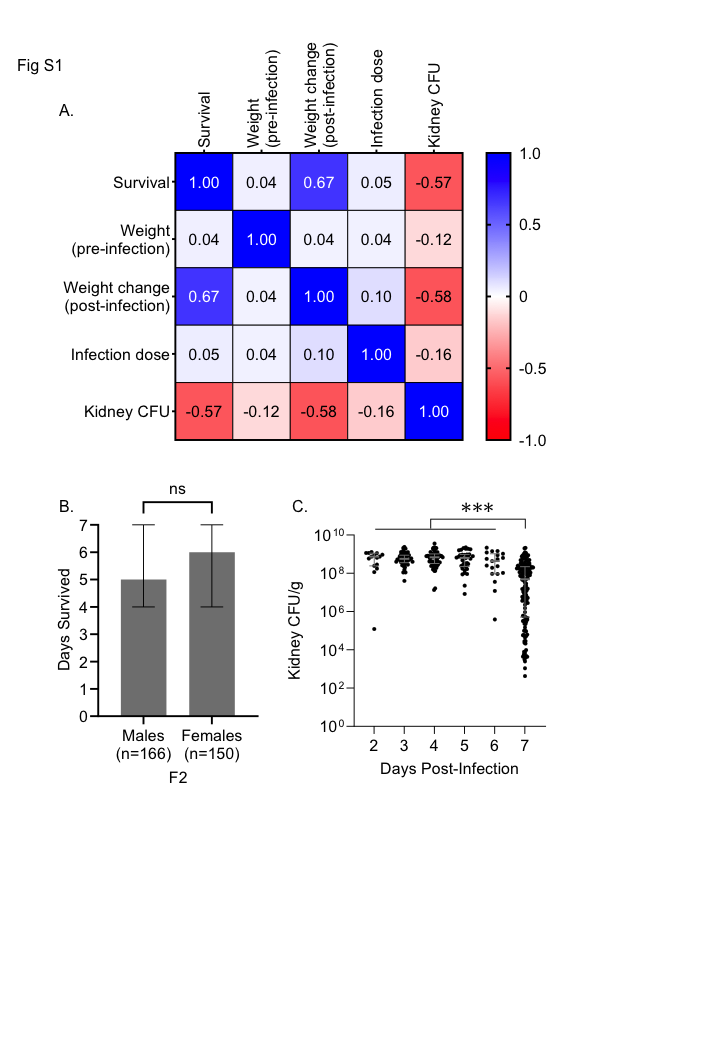

Supplement: S1 Fig — A. Heat map showing Spearman correlation ‘R’ values between survival, weight change, Infection dose, and kidney CFU. B. Survival after infection separated by males and females. C. Kidney colonization of F2 mice by days survive. The median and interquartile range are shown. B–Mann-Whitney and C–Kruskal-Wallis tests were performed (ns- no significance, *** = P < 0.001). (TIFF) [file pgen.1011378.s001.tiff]

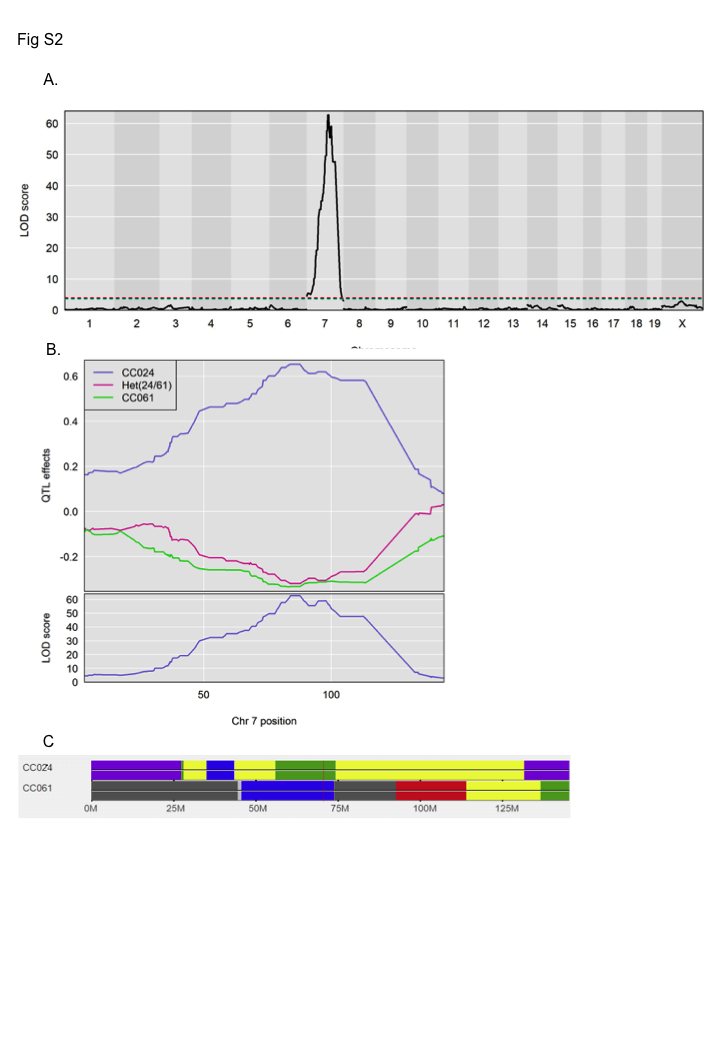

Supplement: S2 Fig — A. LOD plot for coat color after infection (0 –Agouti, 1 –Albino/white). The dotted (Red– 95%, Blue– 90%, Green– 85%) lines represent the significant LOD scores for 999 permutations. B. Founder allele plot for chromosome 7. C. Founder allele contribution for chromosome 7. (TIFF) [file pgen.1011378.s002.tiff]

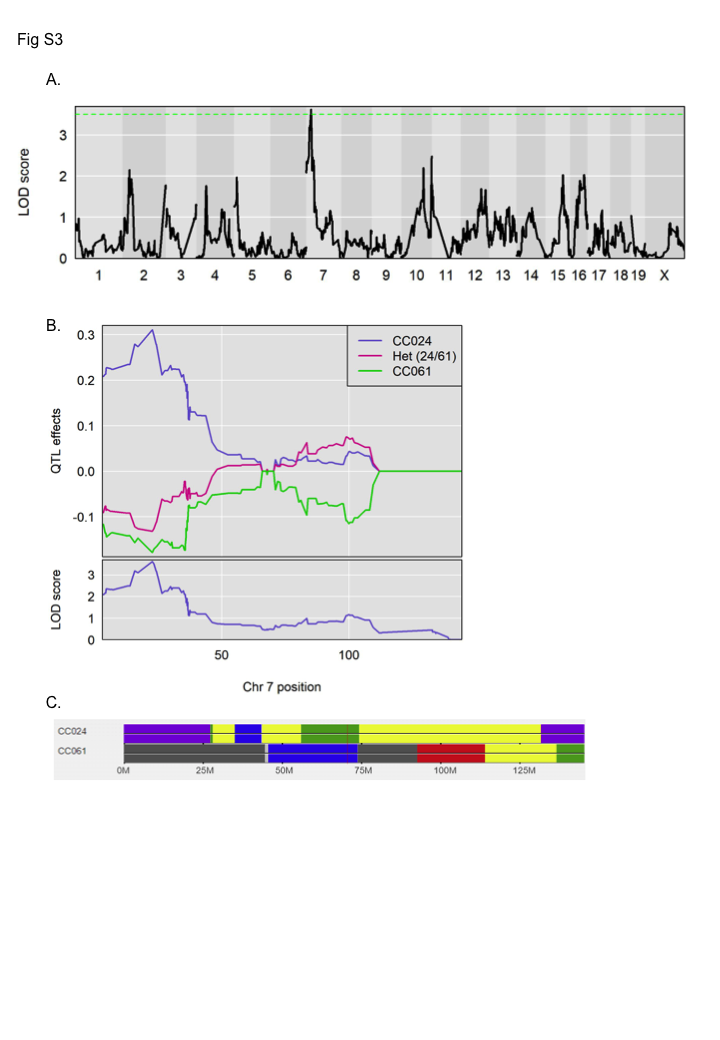

Supplement: S3 Fig — A. LOD plot for rank-transformed weight change after infection. The dotted (Red– 95%, Blue– 90%, Green– 85%) lines represent the significant LOD scores for 999 permutations. B. Founder allele plot for chromosome 7. C. Founder allele contribution for chromosome 7. (TIFF) [file pgen.1011378.s003.tiff]
